# Frequency of Urinary Tract Infections, Gonorrhea, and Chlamydia in Emergency Department Patients With Acute Scrotal Pain

**DOI:** 10.7759/cureus.16347

**Published:** 2021-07-12

**Authors:** Josh Greenstein, Victoria Babson, Jenna Frisolone, Brianna Janiszewski, Samantha Kyvik, Brooke Mason, Michaela Paduch, Tara Igneri, Barry Hahn, Anthony V D'Antoni

**Affiliations:** 1 Emergency Medicine, Staten Island University Hospital, Staten Island, USA; 2 Emergency Medicine, Central Harnett Hospital, Lillington, USA; 3 Trauma Surgery, Jacobi Medical Center, Bronx, USA; 4 Cardiothoracic Surgery, University of Rochester Medical Center, Rochester, USA; 5 Emergency Medicine, Wagner College, New Windsor, USA; 6 Otolaryngology, Jersey Shore University Medical Center, Neptune City, USA; 7 Cardiology, St. Vincent's Medical Center, Bridgeport, USA; 8 Physician Assistant Program, Wagner College, Staten Island, USA

**Keywords:** scrotal pain, urinalysis, uti, sexually transmitted disease, gonorrhea and chlamydia

## Abstract

Background: Acute scrotal pain has many causes. According to the American Urological Association recommendations: history, physical examination, and ultrasound are key in diagnosing acute scrotal pain.

Objective: The primary objective of this study was to evaluate the frequency of urinary tract infections (UTI) on routine Urinalysis (UA) in patients presenting with acute scrotal pain to the emergency department (ED).

Methods: We conducted a multicentered retrospective chart review of patients who presented to the ED with acute scrotal pain. Patient visits from February 1, 2018 to November 1, 2019 from 13 EDs were analyzed. Demographic data, UA interpretation, urine culture, gonorrhea and chlamydia (GC) testing, clinical findings, treatment outcomes, and ultrasounds were recorded. Patients who did not have a UA and scrotal ultrasound performed or who had a diagnosis of scrotal cellulitis or soft tissue infection were excluded.

Results: There were 2,392 patients included in the study. A UTI was present in 173 (7.2%) patients. Of the patients who were found to have a UTI, 100/173 (57.8%) had a concomitant ultrasound diagnosis of epididymitis/orchitis. Also, 731 patients underwent GC testing in addition to standard UA collection, and ultrasound, seven were positive for gonorrhea (0.95%), and 30 were positive for chlamydia (4.10%).

Conclusions: Routine UA collection of patients presenting to the ED with acute scrotal pain should be considered, especially in patients with a concomitant ultrasound diagnosis of epididymo-orchitis. GC testing has limited yield without symptoms suggestive of sexually transmitted infections and a normal ultrasound.

## Introduction

Scrotal complaints are common, making up at least 0.5% of all emergency department (ED) visits each year [1]. The acute scrotum is defined as sudden onset of pain or painful swelling of the scrotum or its contents [2]. There are a number of etiologies that cause acute scrotum, such as testicular torsion, epididymitis, and orchitis. As stated by the American Urological Association: a history, physical examination, and ultrasound (US) are keys in the diagnosis of acute scrotum [3]. In patients presenting to the ED with the acute scrotum, while the scrotal US for some conditions is specific and yields the definitive diagnosis, providers often concomitantly order urinalysis (UA) and gonorrhea and chlamydia (GC) panel as part of the patient evaluation [4]. The literature has shown that both of these tests have many limitations and their role in the diagnosis and management of acute scrotum remains unclear [4]. A cross-sectional study showed that utilization of US, UA, and GC in pediatric males presenting with scrotal pain varies dramatically [5]. In patients with suspected epididymo-orchitis, UA may have a degree of utility, although US is more sensitive and specific for the diagnosis [6]. However, one study found that if US is considered the gold standard for the diagnosis of epididymo-orchitis, the sensitivity of UA is 48.0%, and the specificity is 81.4% [4]. Given the narrow role of UA in the evaluations of scrotal pain, UA may not be necessary for all patients presenting with scrotal pathology [6]. According to the Center for Disease Control, suspected epididymo-orchitis is the only scrotal pathology in which GC testing is indicated in, as it guides treatment [7]. However, many patients tested for GC are empirically treated with antibiotics before results have become available [8]. Additionally, provider judgment for empiric treatment has an overall sensitivity of 67.6% (95%CI 61.8, 73.0) and a specificity of 55% (95%CI 51.7, 58.2) for correct GC detection [8].

The primary objective of this study, was to evaluate the frequency of urinary tract infections (UTI) on routine UA in the assessment of patients presenting to the ED with acute scrotal pain. The secondary objective is to evaluate the frequency of positive GC testing on patients presenting to the ED with scrotal pain.

## Materials and methods

Setting

This was a retrospective multicentered chart review, spanning across 13 EDs within the largest health system in New York, serving approximately 11 million persons in Long Island, Westchester County, and New York City. The EDs included in the study were from tertiary care facilities, community EDs, major metropolitan EDs, as well as a dedicated pediatric hospital.

Population

The study included male patients of all ages, who presented to ED with acute scrotal pain that received UA and the scrotal US from February 1, 2018 to November 1, 2019. The time frame was chosen based on the availability of a common electronic medical record (EMR), which allowed for a comprehensive electronic database allowing for accurate data collection. Exclusion criteria consisted of patients who did not receive a UA, patients with contaminated UA or culture, patients who did not receive a scrotal US, and patients who received a diagnosis of soft tissue infection or cellulitis.

Protocol

Data were obtained on patient encounters through a query of the respective EDs’ EMR. Specifically, scrotal US radiology orders we used to identify patient encounters. Data was then maintained on REDCAP, a secure, web-based application designed to support data capture for research studies. IRB approval was granted by a local institutional review board.

Measurements

Six research staff personnel were trained in the study protocol and data abstraction. A pre-designed, standardized case report form was utilized. A comprehensive chart review was performed, extrapolating many variables for this study. Demographic variables included age, gender, race (Hispanic, not Hispanic, unknown), insurance (private or public), and date of visit. Ages were separated into three ranges: 0-18 years old, 19-35 years old, and greater than 35 years old. These age ranges were created in this manner as the prevalence of certain organisms that may cause epididymo-orchitis varied within these groups and therefore have different treatments. For example, men less than 35 years old are likely to have GC while men greater than 35 years old are likely to have enteric organisms as the etiology [9]. The vital sign of temperature data was classified as either hypothermic defined as <96.4°F, febrile defined as greater than 100.4°F, or normal. Medical history data collected included diabetes mellitus, chronic kidney disease, immunocompromised state, and solitary kidney. The presence of clinical signs and symptoms such as urethral discharge, dysuria, urinary frequency, urinary urgency, flank pain/abdominal pain, rash/discoloration, scrotal tenderness on palpation, and inguinal adenopathy were also part of the data collected. The US diagnosis was formulated following a review of the US radiology impression report, as well as the ED clinical course, discharge diagnosis, and provider documentation. The US diagnosis categories were normal US, epididymo-orchitis, hydrocele/varicocele, testicular torsion, scrotal/testicular mass, and others. A diagnosis of normal US was defined as the US lacking any findings.

Patients who had both epididymo-orchitis and hydrocele on the US were classified as epididymo-orchitis as the authors felt that the hydrocele was likely a reactive process of the epididymo-orchitis. For statistical analysis, categories were created for diagnoses that have similar treatment outcomes. Hydrocele and varicocele were integrated into one category, while orchitis, epididymitis, and epididymitis/orchitis were condensed to another. GC urine test results, urine interpretation, and urine culture results were all identified as positive or negative. There is wide variation in the definition of a positive UA and positive Urine Culture in the literature. Studies have defined a positive UA as requiring 1+ leukocytes or positive nitrites [10,11]. Meanwhile, other studies only required trace leukocytes or positive nitrites for a positive result [12,13]. As a result, for this study, a positive UA was based on the recommendations set forth by the Infectious Disease Society of America (IDSA) [14]. In patients who received both UA and urine culture, the urine culture served as the gold standard to determine if there was an infection in the genitourinary tract. If the patient solely had UA, the UA was considered positive for infection if the urine had positive nitrites or leukocytes of any value, as well as pyuria, which was defined as five white blood cells or greater. A contaminated UA was defined as moderate or more epithelial cells [15]. Urine cultures were considered positive if ≥100,000 CFU/mL [15,16]. A contaminated urine culture was defined by a culture with three or more species of microbes’ growth or was reported contaminated by the lab of the institution [17]. To ensure accurate data collection and appropriate interobserver agreement, all data were reviewed by the lead author (JG) and separately reviewed by other trained study investigators.

Data analysis

A priori power analysis revealed that the minimum sample size needed to achieve significance was 880 subjects (G-power Version 3.1.9.6, Germany, 2016). The data were analyzed using descriptive statistical methods and expressed as frequency counts and percentages for categorical variables or as a mean and standard deviation or median and interquartile range (IQR), as appropriate, for continuous variables. Descriptive statistics were obtained using Spearman Rho Correlations using IBM SPSS Statistics Version 26 (IBM, Inc., Armonk, NY, USA).

## Results

Demographics

There were 2,392 male patients included in this study. The mean age identified was 35.1 years of age with a distribution of 23%, 36.3%, and 40.7%, respectively. The age range of the patients was six weeks to 91 years of age. The majority of patients identified as not Hispanic or Latino (75.5%). As shown in Table 1, 88.2% of the patients had no significant past medical history that would affect overall evaluation. The remaining characteristics of the primary sample are demonstrated in Table 1.

**Table 1:** Demorgraphics characteristics

	Composite		Age					
	n=2,392		0-18, n=550		19-35, n=868		>35, n=974	
Age (mean, IQR)	35.1	29	11.5	12	25.6	31	73.8	49
Sex (n,%)								
Male	2,392	100%	550	23%	868	36.3%	974	40.7%
Primary Insurance (n,%)								
None	181	7.6%	17	9.4%	82	45.3%	82	45.3%
Private	1,295	54.1%	314	24.3%	450	34.7%	531	41.0%
Public	916	38.3%	219	24.0%	336	36.6%	361	39.4%
Secondary Insurance (n,%)								
None	2,034	85.0%	511	25.1%	764	37.6%	759	37.3%
Private	195	8.2%	28	14.4%	51	26.2%	116	59.4%
Public	163	6.8%	11	6.7%	53	32.5%	99	60.8%
Race (n,%)								
Not Hispanic or Latino	1807	75.5%	416	23.0%	612	33.9%	779	43%
Hispanic or Latino	480	20.1%	110	23%	209	43.5%	161	33.5%
Unknown	105	4.4%	24	23%	47	44.8%	34	32.2%
Past Medical History								
None (n,%)	2,109	88.2%	550	26.2%	843	39.9%	716	33.9%
Diabetes Mellitus (n,%)	186	7.8%	0	0%	10	5.4%	176	94.6%
Chronic Kidney Disease (n,%)	28	1.2%	0	0%	2	7%	26	92.9%
Single Kidney (n,%)	4	0.2%	0	0%	1	25%	3	75%
Immunocompromised State (n,%)	65	2.7%	0	0%	12	18.5%	53	81.5%

Symptoms/clinical findings

A summary of specific symptoms and clinical findings is outlined in Table 2. The most common reported symptom in the sample was dysuria in 324 (13.5%) patients. Only 72 (3%) patients were febrile. Palpable scrotal tenderness on examination was found in 1,495 (62.5%) patients. The results demonstrate that the majority of the population did not have symptoms or findings suggestive of a UTI or sexually transmitted infection.

**Table 2:** Symptoms and clinical findings

	Composite		Age					
	n=2,392		0-18, n=550		19-35, n=868		>35, n=974	
Symptoms								
None (n,%)	1,880	78.6%	499	26.5%	698	37.10%	683	36.3%
Discharge (n,%)	40	1.7%	3	7.5%	18	45%	19	47.5%
Dysuria (n,%)	324	13.5%	39	12.0%	114	35.20%	171	52.8%
Urinary Frequency (n,%)	107	4.5%	6	5.6%	31	28.90%	70	65.4%
Urinary Urgency (n,%)	41	1.7%	3	7.3%	7	17.10%	31	75.6%
Exam Findings								
Flank/Abdominal Pain	795	33.2%	123	15.5%	286	36.0%	386	48.5%
Rash/Discoloration	176	7.5%	62	35.2%	32	18.2%	82	46.6%
Scrotal Tenderness	1,495	62.5%	345	23.1%	523	34.9%	627	42.0%
Inguinal Adenopathy	34	1.4%	6	17.7%	12	35.3%	16	47.0%
Temperature								
Normal	2,315	96.7%	537	23.2%	857	37%	921	39.8%
Febrile	72	3.0%	13	18.0%	10	14%	49	68.2%
Hypothermic	5	0.2%	0	0.0%	1	20%	4	80.0%

US diagnoses

The most common US findings are outlined in Table 3. The “other” diagnoses category, which accounted for 0.1% of the diagnoses, included diagnoses such as inguinal hernias, scrotal hematomas, or testicular cysts. Out of the total population, 991 (42%) patients had a normal US finding. Hydrocele/varicocele was the second most common US finding with 716 (30.6%) patients identified, followed by 529 (22.6%) patients with epididymo-orchitis.

**Table 3:** Percentage of ultrasound diagnoses

	Composite		Age					
	n=2,392		0-18, n=550		19-35, n=868		>35, n=974	
Ultrasound Diagnosis								
Normal (n,%)	991	42.0%	281	28.4%	401	40.5%	309	31.1%
Epididymo-orchitis (n,%)	529	22.6%	115	21.8%	121	22.8%	293	55.4%
Hydrocele, Varicocele (n,%)	716	30.6%	90	12.6%	285	39.8%	341	47.6%
Testicular Torsion (n,%)	84	3.4%	71	84.5%	9	10.7%	4	4.8%
Scrotal/Testicular Mass (n, %)	41	1.3%	3	7.3%	14	34.2%	24	58.5%
Other (n,%)	31	0.1%	1	3.2%	27	87.1%	3	96.7%

US diagnoses and urine interpretation

Results between urine interpretation and US diagnoses are shown in Table 4. The most common US diagnosis with a positive UA was epididymo-orchitis, with 100 patients identified (18.9%). Also, 173 (7.2%) patients had a UTI. Of the patients diagnosed with a UTI, 100 (57.8%) patients had an accompanying US diagnosis of epididymo-orchitis.

**Table 4:** US diagnoses and urine interpretation

	Urine Interpretation				
	Composite	Positive		Negative	
	n=2,392	n=173 (7.2%)		n=2,219 (92.8%)	
Ultrasound Diagnosis					
Normal (n,%)	991	37	3.7%	954	96.2%
Epididymo-orchitis (n,%)	529	100	18.9%	429	81.1%
Hydrocele, Varicocele (n,%)	716	34	4.7%	682	95.3%
Testicular Torsion (n,%)	84	2	2.6%	82	97.4%
Scrotal/Testicular Mass (n, %)	41	0	0%	41	100.0%
Other (n,%)	31	0	0%	31	100%

US diagnosis and GC testing result

There were 731 (30.7%) patients who underwent GC testing in addition to standard UA collection. Of those patients, 7 (0.95%) were positive for gonorrhea, and 30 (4.1%) were positive for chlamydia. The most common US finding with a positive GC panel was epididymo-orchitis, with 25 (4.7%) patients identified. The remaining findings of GC testing are outlined in Table 5.

**Table 5:** US diagnoses and gonorrhea/chlamydia testing result

	Gonorrhea Testing Result						Chlamydia Testing Result					
	Total Tested, n=731		Positive, n=7 (0.95%)		Negative, n=7 (0.95%)		Total Tested, n=729		Positive, n=30 (4.1%)		Negative, n=699 (95.9%)	
Ultrasound Diagnosis												
Normal (n,%)	273	27.5%	2	0.2%	271	27.3%	271	27.4%	5	0.5%	266	26.8%
Epididymo-orchitis (n,%)	203	38.4%	3	0.3%	200	37.8%	204	38.6%	22	4.0%	182	34.4%
Hydrocele, Varicocele (n,%)	230	32.0%	2	0.2%	228	31.8%	229	31.9%	3	0.4%	226	31.6%
Testicular Torsion (n,%)	12	14.3%	0	0.0%	12	14.3%	12	14.3%	0	0.0%	12	14.3%
Scrotal/Testicular Mass (n, %)	9	22.0%	0	0.0%	9	22.0%	9	22.0%	0	0.0%	9	22%
Other (n,%)	4	12.9%	0	0.0%	4	12.9%	4	13.0%	0	0.0%	4	13%

## Discussion

This study population had a mean age of 35.1 years, with the majority, 88.2%, having no significant contributory past medical history. The most common finding on US was a normal US finding in 42% of patients, followed by hydrocele/varicocele in 30.6% of patients and epididymo-orchitis in 22.6% of patients. A positive UA was identified in 7.2% of patients. The most common US diagnosis with a positive UA was epididymo-orchitis in 18.9% of patients. Of the 734 patients (30.7%) who underwent GC testing, in addition to standard UA collection, 0.95% were positive for gonorrhea, and 4.1% were positive for chlamydia. The most common US finding with a positive GC panel was epididymo-orchitis in 4.7% of patients.

There are limited studies that have examined the utility of UA and GC testing in patients presenting with acute scrotal pain. Previous studies have focused solely on the acute scrotum in pediatric populations [15,16], ​​​​​​in adult populations [18,19], and included a much smaller number of patients. The inclusion of data from multiple sites, with a wide age range, and a large spectrum of acute scrotal etiologies, allows for greater generalizability of the results to the study. As seen in the demographic data, the majority of the study population had no significant comorbidities and had similar clinical presentations, as well as exam findings. These characteristics provide a clear picture of the typical patient presenting with acute scrotum to the ED and allow the findings to be applied to the general population.

A similar prior study evaluated the utility of the UA in pediatric and adult patients with epididymo-orchitis [6]. It was reported that of the 69.1% of patients with an acute scrotum that had UA done, and 26.7% were positive. Our study had a similar population of both adult and pediatric age groups. The study showed a similar but smaller percentage of 18.9% of patients with epididymo-orchitis having a positive UA. This difference could be accounted for by the fact our study included all etiologies of the acute scrotum, not just epididymo-orchitis. Another prior study by Kim et al. looked at urine culture growth in adult patients with epididymo-orchitis [18]. Data showed that patients with acute epididymitis had a positive urine culture 30.8% of the time. The urine culture revealed that sexually transmitted infections such as Chlamydia trachomatis or Neisseria gonorrhoeae were found in 65% of these patients. The remainder of the sample, or 45%, had gram-negative enteric bacteria (non-STIs), including Escherichia coli, Staphylococcus aureus, S. epididymis, or Enterococcus faecalis. These results highly differ from our study, as the majority of patients had a sexually transmitted etiology of epididymo-orchitis. Our study had a small percentage of positive GC panels (0.95% for gonorrhea and 4.1% for chlamydia). The few patients in our study with symptoms suggestive of a sexually transmitted infection could contribute to the differences found in the frequency of causative organisms. Additionally, some providers empirically treated for GC and did not undergo GC testing. In the study done by Kim et al., 90% of patients with a positive urine culture, and 11% of patients with a negative urine culture, had urethral discharge, as compared with our study, where only 1.7% of the whole population had discharged. Our study is consistent with prior literature that demonstrated that UA and urine culture are not positive in the majority of patients with scrotal pain, although it is still considered part of the workup for acute scrotal pain [20].

Clinical judgment should be utilized since 7.2% of patients had a UTI, and 5.1% of patients had a positive GC test in this study. Additionally, the results of our study suggest that GC testing should be utilized in patients with risk factors and symptoms suggestive of sexually transmitted infections. Furthermore, GC testing can help differentiate whether a sexually transmitted infection or gram-negative enteric bacteria was the cause of epididymo-orchitis.

Since this study was exploratory, the final answer to the diagnostic utility of UA remains unanswered. However, future implications can expand on this knowledge and may determine the sensitivity and specificity of UA, urine culture, and GC testing in different scrotal etiologies. Furthermore, this research clinically can help formulate an algorithm for the evaluation of acute scrotum and to determine which patients would likely benefit from UA and GC testing in the ED.

Limitations

This study is a retrospective chart review, therefore, subject to the limitations of such studies. In addition, the results of this study may be influenced or limited by the lack of documentation that is inherent to a retrospective chart study. Furthermore, it is possible that occasionally we were not able to fully visualize the clinical picture, in turn, interpreting UA without a culture difficult. Moreover, it is possible that some UAs were interpreted and attributed as positive UTI when they could have been caused by a GC infection. Moreover, there is no universally accepted definition of a UTI based on a UA sample alone, and some patients included in the study lacked a urine culture. As a result, correct interpretation of UTI may have been affected. However, the authors chose to define a UTI based on the IDSA guidelines, which resulted in a set of criteria to diagnose a positive UTI that was specific but less sensitive to mitigate this limitation. The definition of a positive UA used in this study likely resulted in the inclusion of false-positive UTIs. Lastly, only 30% of patients underwent GC testing, thus limiting the extent to which conclusions can be drawn.

## Conclusions

In this study, 7.2% of patients had a UTI, and the most common US diagnosis with a positive UA was epididymo-orchitis in 18.9% of patients. However, only 5.1% had a positive GC result. Routine UA collection of patients presenting to the ED with acute scrotal pain should be considered, especially in patients with a concomitant US diagnosis of epididymo-orchitis. Additionally, GC testing has limited yield without symptoms suggestive of sexually transmitted infections and a normal US.
